# Characteristics of hematological parameters on admission in COVID-19 Omicron variant infected in Chinese population: a large-scale retrospective study

**DOI:** 10.1186/s12879-023-08771-2

**Published:** 2023-11-27

**Authors:** Wei Xia, Tao Jiang, Yafeng Tan, Chengbin Li, Song Wu, Bing Mei

**Affiliations:** Department of Laboratory Medicine, Jingzhou Hospital Affiliated to Yangtze University, Jingzhou, Hubei 434020 China

**Keywords:** Hematological parameters, Coronavirus disease 2019, Omicron variant, Pulmonary Infection

## Abstract

**Background:**

The Omicron variant of SARS-CoV-2, currently the most prevalent strain, has rapidly spread in Jingzhou, China, due to changes in the country’s epidemic prevention policy, resulting in an unprecedented increase in cases. Previous studies reported hematological parameters’ predictive value in COVID-19 severity and prognosis, but their relevance for early diagnosis in patients infected by the Omicron variant, particularly in high-risk pneumonia cases, remains unclear. Our study aimed to evaluate these parameters as early warning indicators for Omicron-infected patients in fever clinics and those with pulmonary infections (PI).

**Methods:**

A total of 2,021 COVID-19 patients admitted to the fever clinic and infectious disease department of Jingzhou Hospital Affiliated to Yangtze University from November 1, 2022, to December 31, 2022, were retrospectively recruited. Demographic and hematological parameters were obtained from the electronic medical records of eligible patients. These hematological parameters were analyzed by receiver operating characteristic (ROC) curves to determine whether they can be used for early diagnosis of COVID-19 patients in fever clinics and the presence of PI in COVID-19 patients.

**Results:**

Statistical differences in hematological parameters were observed between COVID-19 patients with fever and PI and control groups (P < 0.01). The ROC curve further demonstrated that lymphocyte (LYM) counts, neutrophil (NEU) counts, monocyte-to-lymphocyte ratios (MLR), platelet-to-lymphocyte ratios (PLR), white blood cell counts (WBC), and mean corpuscular hemoglobin concentration (MCHC) were the top 6 indicators in diagnosing Omicron infection with fever, with area under the curve (AUC) values of 0.738, 0.718, 0.713, 0.702, 0.700, and 0.687, respectively (P < 0.01). Furthermore, the mean platelet volume (MPV) with an AUC of 0.764, red blood cell count (RBC) with 0.753, hematocrit (HCT) with 0.698, MLR with 0.694, mean corpuscular hemoglobin (MCH) with 0.676, and systemic inflammation response indexes (SIRI) with 0.673 were the top 6 indicators for the diagnosis of COVID-19 patients with PI (P < 0.01).

**Conclusions:**

LYM, NEU, MLR, PLR, WBC, and MCHC can serve as potential prescreening indicators for Omicron infection in fever clinics. Additionally, MPV, RBC, HCT, MLR, MCH, and SIRI can predict the presence of PI in COVID-19 patients infected by the Omicron variant.

## Background

Coronavirus disease 2019 (COVID-19), attributed to severe acute respiratory syndrome coronavirus 2 (SARS-CoV-2), was first observed in December 2019. It has since spread across numerous countries globally, detrimentally affecting the national economy and the healthcare system [1, 2]. As of April 12th, 2023, the World Health Organization (WHO) had documented 762,791,152 cases of confirmed COVID-19 and 6,879,025 reported deaths from COVID-19. Subsequently, the WHO has identified several novel variants of concern (VOC) of the SARS-CoV-2 virus, including Alpha, Beta, Gamma, and Delta, which have consecutively been detected in numerous countries [3]. The Omicron variant, B.1.1.529, was initially identified in South Africa and Botswana. It was officially recognized as the fifth VOC by the WHO in November 2021 [4]. Several studies have demonstrated that the Omicron variant, which has the highest number of viral structural protein mutations to date, has increased transmission, risk of reinfection, and immune escape compared to the original wild-type strain and the other four VOCs [5–8]. Moreover, the Omicron variant is currently the most highly transmissible strain, and has become a significant epidemic strain worldwide, sparking the fourth wave of the global COVID-19 pandemic [9, 10]. Fortunately, the clinical manifestation of the Omicron variant of SARS-CoV-2 infection is predominantly upper respiratory infection or asymptomatic, resulting in very low hospitalization and death risks [11–13]. In December 2022, a change in China’s epidemic prevention policy triggered a surge in cases of the SARS-CoV-2 Omicron variant, which quickly became the dominant circulating strain. This led to an unprecedented and rapid spread of the virus in the Hubei, Jingzhou regions of China.

Several research studies have indicated that hematological parameters can serve as a reliable indicator of the severity and prognosis of immune-related diseases, such as bloodstream infections (BI) [14], cardiovascular diseases [15], sepsis [16], diabetic kidney disease (DKD) [17], and cancer [18]. Dysregulated immune response and hyperinflammatory state are common causes of mortality in severely affected COVID-19 patients [19]. Furthermore, the SARS-CoV-2 Omicron variant also can trigger an excessive immune response known as the cytokine storm, which leads to the production of proinflammatory cytokines. This response can significantly raise the risk of patients developing acute respiratory distress syndrome (ARDS) or multiple organ dysfunction syndrome (MODS), both of which can result in serious health problems and even death [20, 21]. Additionally, previous studies have shown that the presence of hematologic abnormalities in COVID-19 has been linked with the progression and severity of the disease and mortality [22, 23]. Similarly, indexes of leukocytes and systemic inflammation on admission, including the derived neutrophil to lymphocyte ratio (dNLR), systemic immune-inflammation index (SII), neutrophil to lymphocyte ratio (NLR), monocyte to lymphocyte ratio (MLR), platelet to lymphocyte ratio (PLR), and systemic inflammatory response index (SIRI), can predict the severity of COVID-19 infection and in-hospital mortality in patients [24–26].

Although some studies have confirmed that the Omicron variant has a lower prevalence of pneumonia than other strains and primarily targets the upper respiratory tract, this does not imply that the Omicron variant lacks clinical pathogenicity [27, 28]. A previous study found that using cost-effective complete blood parameters and their derived hematological profiles can predict the clinical conditions of patients with COVID-19 variants, as well as their need for hospitalization or ICU admission [29]. During the early stages of the COVID-19 pandemic with the wild-type variant, our research team focused on less costly complete blood parameters to assess the clinical value of these indicators in patients at high risk of severe cases [41]. However, the value of hematological parameters in COVID-19 patients infected with the SARS-CoV-2 Omicron variant remains unclear, and screening for patients at risk of pneumonia from Omicron infection is limited. In the Omicron era, depending solely on RT-qPCR for COVID-19 detection presents challenges like lengthy turnaround times, high false-negative rates, and increased costs, leading to wasted medical resources and treatment delays [30]. Rapid, affordable, and widely accessible biomarkers are essential for early detection of those at risk of pneumonia, enabling rational intervention. Therefore, it’s necessary to further determine which of the routine hematological parameters can be relied upon to predict the severity of Omicron variant cases. Additionally, limited studies have not thoroughly investigated the predictive value of hematological parameters for differentiating SARS-CoV-2 Omicron variant-infected patients from the virus negative individuals.

According to a national study conducted by the Chinese Center for Disease Control and Prevention (CDC), between September 26, 2022, and January 30, 2023, a total of 20,582 valid SARS-CoV-2 genome sequences were identified nationwide from domestic cases, all of which predominantly were Omicron variants with a total of 73 lineages. During the same period, Omicron variants of BA.5.2 and its sub-lineages were predominant in the Hubei Province of China [63]. Considering the patients in this study were enrolled cases between November 1 and December 31, 2022, we can easily infer that during this period, the Omicron variant was the most common and prevalent variant of SARS-CoV-2 in the city of Jingzhou, Hubei province. Accordingly, present investigation endeavors to evaluate predictive value of hematological parameters and derived hematological profiles on admission, including NLR, dNLR, PLR, MLR, SII, and SIRI, in early distinguishing SARS-CoV-2 Omicron variant infected patients with fever and combined with pulmonary infection during the coronavirus pandemic outbreak in Jingzhou, Hubei, China.

## Methods

### Study participants

**Fig. 1:**
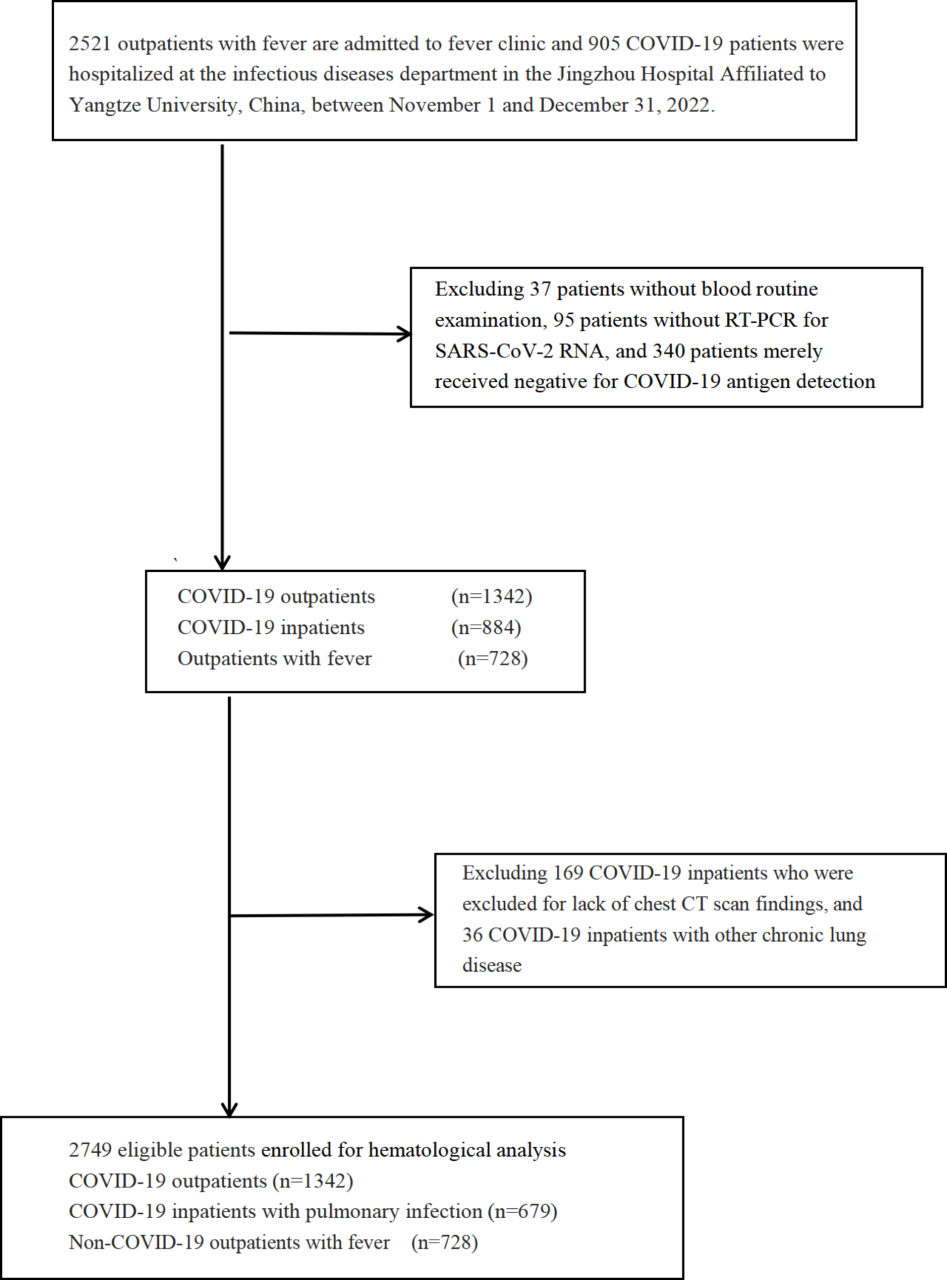
Flow chart of this study

As the Omicron variant of SARS-CoV-2 gradually evolves into a highly transmissible but relatively less pathogenic variant, which increases the difficulty of COVID-19 prevention and control measures. The national epidemic situation has shown multiple scattered infected cases all over the China, as of October 2022. The Chinese government initiated a transition from the optimization of China’s “dynamic COVID-zero strategy” in November 2022 to a gradual relaxing of COVID-19 prevention and control measures in December 2022, which directly resulted in an unprecedented increase in the infection rate in the city of Jingzhou, Hubei province [64, 65]. A similar study confirmed that the infection rate reached its peak between December 19 and 21, 2022, with 82.4% of the Chinese population infected as of February 7, 2023 [66]. Additionally, this period naturally reflects the process of the Omicron variant of SARS-CoV-2 infecting the local population, from a lower infection period to a peak infection period. In this retrospective study, we recruited a total of 3426 patients between November 1 and December 31, 2022. Of these patients, 2521 were fever patients treated at the fever clinic and 905 patients tested positive for COVID-19 and were admitted to the infectious disease department at Jingzhou Central Hospital. Following the National COVID-19 Diagnosis and Treatment Protocol (Tenth Edition), all positive patients for the SARS-CoV-2 Omicron variant were diagnosed by real-time reverse transcription polymerase chain reaction (RT‒PCR) or COVID-19 antigen detection. The inclusion criteria were as follows: (1) individuals were diagnosed with COVID-19 through either RT-qPCR or antigen detection methods in accordance with the protocol for the National COVID-19 Diagnosis and Treatment (Tenth Edition) (2) individuals initially diagnosed in our hospital. Moreover, patients who meet any of the following criteria were ineligible: (1) lack of complete blood count (CBC) test; (2) lack of chest computed tomography (CT) scan; (3) incorporated subjects who merely received negative for COVID-19 antigen detection; (4) individuals without RT-qPCR for SARS-CoV-2 RNA; (5) having other lung infection or disease or pulmonary malignancies. We designed a control group with negative fever patients for SARS-CoV-2 by RT-qPCR tests in fever clinics. Furthermore, current study subjects were categorized into two groups based on whether they had or did not have pulmonary infection (PI): the non-PI group, consisting of 1342 cases, and the PI group, consisting of 679 cases. This study design flowchart is illustrated in Fig. 1.

### Measurements of hematological parameters

We obtained demographic and hematological data from our hospital’s electronic medical records, including age, gender, categorical age value, and hematological parameters. We enrolled hematological parameters from patients on admission and analyzed them to prevent the drug’s effects on these variables. In this study, an automated hematology analyzer (XN-3000, Sysmex, Japan) was used to perform a routine complete blood count on admission of the study participants. The hematological parameters that were measured and documented included white blood cell (WBC) count, neutrophil (NEU) count, lymphocyte (LYM) count, monocyte (MON) count, red blood cell count (RBC), hemoglobin (HGB), hematocrit (HCT), mean corpuscular volume (MCV), mean corpuscular hemoglobin (MCH), mean corpuscular hemoglobin concentration (MCHC), red cell volume distribution width-coefficient of variation (RDW-CV), platelet (PLT) count, mean platelet volume (MPV), platelet hematocrit (PCT), and platelet distribution width (PDW). Additionally, derived hematological profiles were calculated as follows: derived neutrophil-to-lymphocyte ratio (dNLR), neutrophil count/(white blood cell count - neutrophil count); neutrophil-to-lymphocyte ratio (NLR); platelet-to-lymphocyte ratio (PLR), and the (MLR) monocyte-to-lymphocyte ratio. Moreover, the systemic inflammatory index (SII), platelet count × neutrophil count / lymphocyte count, and the systemic inflammation response index (SIRI), monocyte count× neutrophil count/ lymphocyte count.

### Statistical analysis

The Shapiro‒Wilk test was performed to assess variable distribution. Normally distributed variables were reported as mean ± standard deviation (SD) and analyzed by the independent student’s t-test, while skewed distributed variables were presented as median (interquartile range, IQR) and analyzed by the nonparametric Mann-Whitney U test to compare differences between two groups. Categorical variables were reported as frequency or percentage (%) and compared between two groups using the chi-square test or Fisher’s exact test. Pearson’s correlation coefficient was calculated for analyzing association between fever days and hematological parameters. No imputations were performed for missing data, as the percentage of missing data was less than 5% for all outcomes [31]. Receiver-operating characteristic (ROC) analysis was performed to determine the discriminatory power of hematological parameters, and the area under the curve (AUC), optimal cut-off value, sensitivity, and specificity respectively was calculated. Statistical analysis and mapping were performed using MedCalc software (version 20.01) and SPSS software (version 26.0). P values < 0.05 were considered statistically significant.

## Results

**The analysis of the sequencing chart of SARS-CoV-2 variants during the between October 24, 2022 and January 2, 2023 in Chinese population**.

**Fig. 2:**
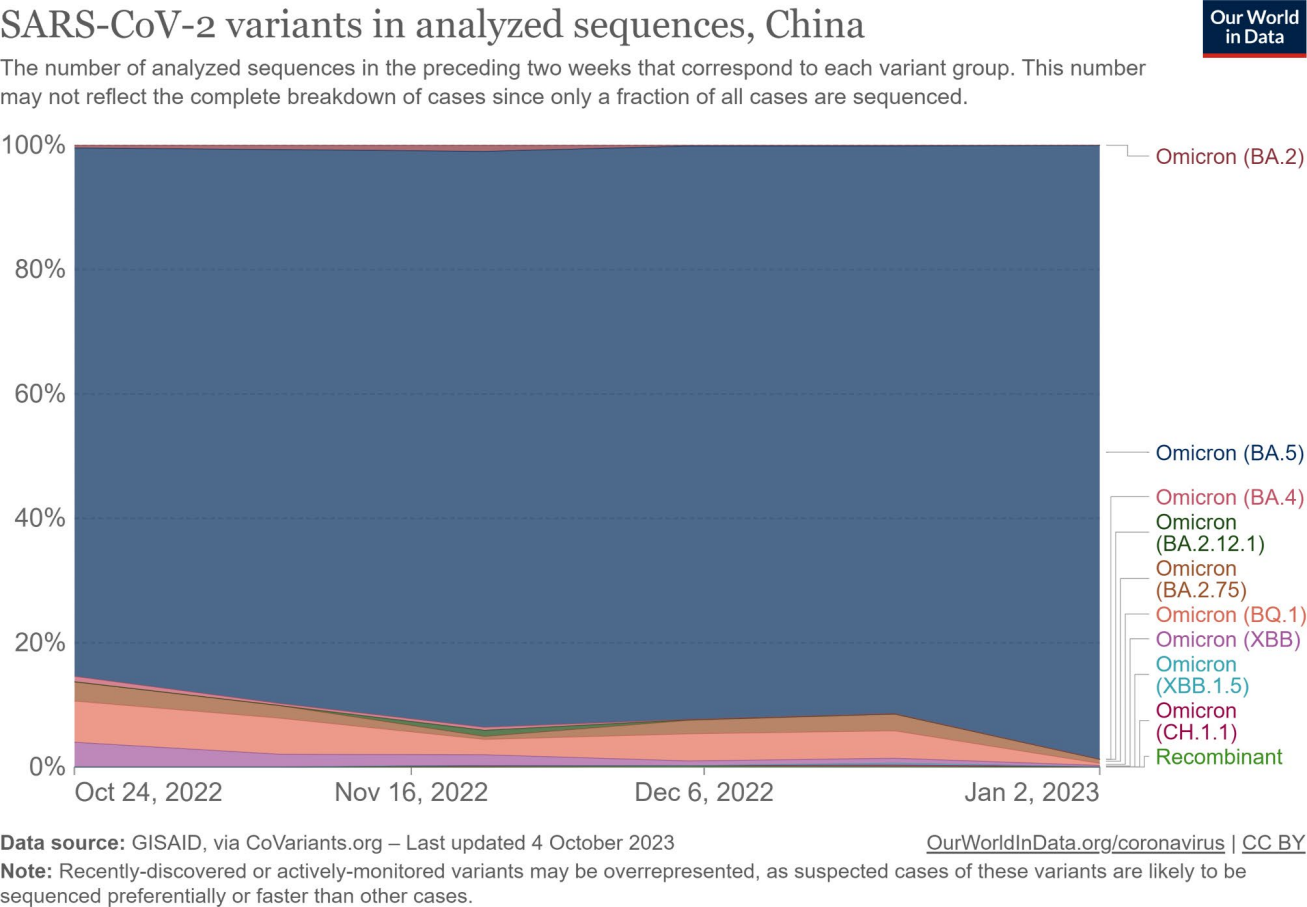
The analysis of the sequencing chart of SARS-CoV-2 variants during the between October 24, 2022 and January 2, 2023 in Chinese population

As indicated in Fig. 2, by using public databases of sequenced sourced from, and detailed website links as follow: https://ourworldindata.org/grapher/covid-variants-area?time=2022-10-24..2023-09-25&country=~CHN. The results showed that during the selected period of our study, the prevalence of SARS-CoV-2 variants in the Chinese population was predominantly attributed to the Omicron variant, accounting for nearly 100% of the observed cases, which provides rationality of the current study.

### Demographics and hematological characteristics of patients in the fever clinic

A total of 2070 fever patients admitted to the Jingzhou Hospital Affiliated to Yangtze University of fever clinic between November 1 and December 31, 2022, were recruited for the study, based on their demographics and hematological parameters as illustrated in Table 1. Of all the patients, the median age was 20 years (interquartile range (IQR): 11–37 years); 885 patients (42.75%) were aged < 18 years, 1022 patients (49.37%) were aged 18–60 years, and 163 patients (7.87%) were aged ≥ 60 years. Furthermore, the majority of fever patients (1064/2070, 51.40%) and 728 diagnosed COVID-19 patients (54.25%) were predominantly female.

**Table 1:** Demographics and hematological parameters of all patients with fever

Variables	Total patients	Control group	COVID-19 patients with fever	P-value
No, (%)	2070	728 (35.17)	1342 (64.83)	
Age, years	20 (11, 37)	15 (5, 24)	26 (14,41)	**< 0.01**
Gender, N (%)				**< 0.01**
Female	1064 (51.40)	336 (46.15)	728 (54.25)	
Male	1006 (48.60)	392 (53.85)	614 (45.75)	
Categorical of ages, n (%)				
< 18 years	885 (42.75)	412 (56.59)	473 (35.25)	0.59
18–60 years	1022 (49.37)	277 (38.04)	745 (55.51)	**< 0.01**
≥ 60 years	163 (7.87)	39 (5.36)	124 (9.24)	**0.01**
Hematological profiles				
WBC counts, ×10^9^/L	6.49 (5.03, 8.59)	8.18 (5.82, 10.93)	5.97 (4.72, 7.57)	**< 0.01**
Neutrophils count, ×10^9^/L	4.86 (3.41, 6.76 )	5.89 (3.76, 8.55)	4.51 (3.28,5.97)	**< 0.01**
Lymphocytes count, 10^9^/L	0.84 (0.54, 1.33)	1.21(0.80, 1.95)	0.70 (0.48, 1.06)	**< 0.01**
Monocyte count, ×10^9^/L	0.53 (0.40, 0.70)	0.57 (0.40, 0.74)	0.51 (0.39, 0.68)	**< 0.01**
RBC counts, ×10^12^/L	4.63 (4.32, 4.95)	4.58 (4.30, 4.88)	4.65 (4.33, 4.98)	**0.01**
HGB, g/L	136.00 (126.00, 147.00)	134.00 (126.00, 145.00)	136.00 (127.00, 148.00)	**0.01**
HCT, %	40.30 (37.90, 43.40)	39.50 (37.10, 42.00)	40.70 (38.30, 44.00)	**< 0.01**
MCV, fl.	88.20 (84.70, 91.50)	87.00 (83.40, 90.60)	88.80 (85.60, 92.00)	**< 0.01**
MCH, pg	29.80 (28.30, 31.10)	29.70 (28.30, 31.40)	29.80 (28.40, 31.00)	0.35
MCHC, g/L	337.00 (330.00, 344.00)	342.00 (334.00, 351.00)	336.00 (329.00, 341.00)	**< 0.01**
RDW-CV, %	12.50 (12.10, 13.00)	12.60 (12.20, 13.10)	12.50 (12.10, 13.00)	**0.01**
Platelet counts, ×10^9^/L	197.00 (165.00, 238.00)	209.00 (175.00, 255.00)	193.00 (158.00, 228.00)	**< 0.01**
MPV, fl.	9.20 (8.50, 9.90)	8.90 (8.30, 9.70)	9.30 (8.60, 10.00)	**< 0.01**
PCT,%	18.20 (15.50, 21.10)	18.80 (16.10, 21.80)	18.00 (15.20, 20.60)	**< 0.01**
PDW, %	15.90 (15.70, 16.20)	15.90 (15.60, 16.20)	16.00 (15.70, 16.20)	**< 0.01**
Derived hematological profiles				
dNLR	3.32 (1.97, 5.12)	2.98 (1.67, 5.03)	3.45 (2.13, 5.19)	**< 0.01**
NLR	5.88 (3.00, 10.27)	4.66 (2.28, 8.86)	6.48 (3.43, 10.94)	**< 0.01**
PLR	227.37 (145.30, 352.73)	170.30 (110.53, 259.04)	264.18 (173.15, 400.00)	**< 0.01**
MLR	0.61 (0.39, 0.96)	0.43 (0.28, 0.65)	0.72 (0.48, 1.07)	**< 0.01**
SII	1109.11 (585.27, 1985.28)	967.38 (510.47, 1838.21)	1190.39 (628.41, 2096.84)	**< 0.01**
SIRI	3.00 (1.54, 5.68)	2.63 (1.20, 5.20)	3.23 (1.75, 5.88)	**< 0.01**

**Abbreviations**: COVID-19, 2019-coronavirus disease; WBC, white blood cell count; RBC, red blood cell count; HGB, hemoglobin; HCT, hematocrit; MCV, mean corpuscular volume; MCH, mean corpuscular hemoglobin; MCHC, mean corpuscular hemoglobin concentration; RDW-CV, red cell volume distribution width-coefficient of variation; MPV, mean platelet volume; PCT, plateletcrit; PDW, platelet distribution width; dNLR, derived neutrophil to lymphocyte ratio; PLR, platelet-to-lymphocyte ratio; MLR, monocyte -to-lymphocyte ratio; NLR, neutrophil -to- lymphocyte ratio; SII, systemic immune-inflammation index; SIRI, systemic inflammation response index

**Note**: The control group is defined as SARS-CoV-2 negative patients with fever. For categorical variables, frequencies and percentages are reported. For continuous variables, data are presented as either mean ± standard deviation (SD) for normally distributed data or median and interquartile range (IQR) for non-normally distributed data. P values < 0.05 were considered statistically significant and are denoted in bold

We further divided all patients with fever into two groups: SARS-CoV-2 negative patients with fever (728 cases, 35.17%) were the control group, and COVID-19 patients with fever (1342 cases, 64.83%) were diagnosed by RT-qPCR tests or antigen detection that indicated SARS-CoV-2 presence. Of them, the median age was 15 years (IQR: 5–24 years) in the control group, and the median age was 26 years (IQR: 14–41 years) in the COVID-19 patients with fever group. There were 745 COVID-19 patients with fever (55.51%) aged 18–60 years and 124 patients with fever (9.24%) aged ≥ 60 years. This difference was statistically significant (p < 0.05). There was a significant difference in gender distribution between the two groups (p < 0.01). In the control group, 46.15% of patients were female, while in the COVID-19 patients with fever group, the proportion of females was 54.25% (p < 0.01).

We enrolled 728 fever patients who tested negative for SARS-CoV-2 as the controls and compared hematology parameters with those of COVID-19 patients with fever, as detailed in Table 1, the results indicated a significant decrease in WBC counts, NEU counts, LYM counts, MON counts, MCHC, RDW-CV, PLT counts, and PCT in COVID-19 fever patients compared to the control group (p < 0.01). Conversely, COVID-19 patients with fever exhibited significantly higher levels of RBC counts, HGB, HCT, MCV, MPV, PDW, dNLR, PLR, MLR, NLR, SII, and SIRI levels compared to the control group (p < 0.01). Additionally, we found no statistically significant differences between the control group and COVID-19 patients with fever in terms of MCH and the categorical of ages < 18 years (p > 0.05). Taken together, the above findings show that both SARS-CoV-2 negative patients with fever and COVID-19 patients infected with the Omicron variant exhibit abnormalities in hematological parameters.

### Demographic and hematological characteristics of Omicron variant infected COVID-19 patients

During November 1 and December 31, 2022, in our hospital medical workers admitted 2021 individuals diagnosed with the COVID-19. As shown in Table 2, the median age of all the patients was 38 years (IQR: 18–64 years). Among the patients, 488 (24.15%) were aged < 18 years, 951 (47.06%) were aged 18–60 years, and 582 (28.80%) were aged ≥ 60 years. In terms of gender distribution, there were 1003 male patients (49.63%) and 1018 female patients (50.37%). Additionally, we further separated all COVID-19 patients as follows: non-pulmonary infection group (1342 cases, 66.40%) and pulmonary infection group (679 cases, 33.60%) were individuals determined by imaging characteristics of abnormal chest CT scans. Of them, patients in the non-pulmonary infection (PI) group had a median age of 26 years (IQR: 14–41 years), while patients in the PI group had a median age of 68 years (IQR: 56–76 years). Specifically, 15 patients (2.21%) were < 18 years, 206 patients (30.34%) were between 18 and 60 years, and 458 patients (67.45%) were ≥ 60 years. The difference in age distribution between the two groups was statistically significant (p < 0.01). Statistical differences were observed in the gender proportion of the two groups (p < 0.01): 54.25% in the non-PI group with 728 females patients and 42.71% in the PI group with 290 females patients (p < 0.01).

**Table 2:** Demographics and hematological parameters of all COVID-19 patients with or without pulmonary infection

Variables	Total patients	Non-pulmonary infection group	Pulmonary infection group	P-value
No, (%)	2021	1342 (66.40)	679 (33.60)	
Age, years	38.00 (18.00, 64.00)	26.00 (14.00, 41.00)	68.00 (56.00, 76.00)	< 0.01
Gender, N (%)				< 0.01
Female	1018 (50.37)	728 (54.25)	290 (42.71)	
Male	1003 (49.63)	614 (45.75)	389 (57.29)	
Categorical of ages, n (%)				
< 18 years	488 (24.15)	473 (35.25)	15 (2.21)	< 0.01
18–60 years	951 (47.06)	745 (55.51)	206 (30.34)	< 0.01
≥ 60 years	582 (28.80)	124 (9.24)	458 (67.45)	< 0.01
Hematological profiles				
WBC counts, ×10^9^/L	5.75 (4.49, 7.45)	5.97 (4.72, 7.57)	5.32 (3.98, 7.12)	< 0.01
Neutrophils count, ×10^9^/L	4.25 (2.96, 5.80)	4.51 (3.28, 5.97)	3.66 (2.49,5.34)	< 0.01
Lymphocytes count, 10^9^/L	0.78 (0.51, 1.16)	0.70 (0.48, 1.06)	0.92 (0.64, 1.32)	< 0.01
Monocyte count, ×10^9^/L	0.49 (0.36, 0.65)	0.51 (0.39, 0.68)	0.44 (0.31,0.59)	< 0.01
RBC counts, ×10^12^/L	4.50 (4.15, 4.87)	4.65 (4.33, 4.98)	4.16 (3.85, 4.50)	< 0.01
HGB, g/L	134.00 (123.00, 145.00)	136.00 (127.00, 148.00)	127.00 (117.00, 139.00)	< 0.01
HCT, %	40.00 (37.10, 43.10)	40.70 (38.30, 44.00)	37.70 (35.10, 40.90)	< 0.01
MCV, fl.	89.70 (86.50, 92.60)	88.80 (85.60, 92.00)	90.90 (88.30, 94.30)	< 0.01
MCH, pg	30.20 (28.80, 31.30)	29.80 (28.40, 31.00)	30.80 (29.80, 31.90)	< 0.01
MCHC, g/L	336.00 (330.00, 342.00)	336.00 (329.00, 341.00)	338.00 (333.00, 344.00)	< 0.01
RDW-CV, %	12.60 (12.20, 13.10)	12.50 (12.10, 13.00)	12.80 (12.30, 13.30)	< 0.01
Platelet counts, ×10^9^/L	191.00 (152.00, 232.00)	193.00 (158.00, 228.00)	183.00 (138.00, 246.00)	0.01
MPV, fl.	9.77 ± 1.26	9.39 ± 1.07	10.53 ± 1.25	< 0.01
PCT,%	18.92 ± 6.04	18.20 ± 4.38	20.34 ± 8.24	< 0.01
PDW, %	15.74 ± 1.36	16.00 ± 0.40	15.23 ± 2.19	< 0.01
Derived hematological profiles				
dNLR	3.09 (1.92, 4.86)	3.45 (2.13, 5.19)	2.46 (1.67, 3.92)	< 0.01
NLR	5.47 (2.97, 9.88)	6.48 (3.43, 10.94)	3.89 (2.41, 6.97)	< 0.01
PLR	241.28 (157.66, 370.21)	264.18 (173.15. 400.00)	193.20 (135.06, 294.44)	< 0.01
MLR	0.62 (0.41, 0.97)	0.72 (0.48, 1.07)	0.46 (0.31, 0.69)	< 0.01
SII	1005.33 (532.18, 1898.00)	1190.39 (628.41, 2096.84)	721.17 (400.18, 1439.13)	< 0.01
SIRI	2.62 (1.32, 5.21)	3.23 (1.75, 5.88)	1.63 (0.83, 3.32)	< 0.01

**Abbreviations**: COVID-19, 2019-coronavirus disease; PI, pulmonary infection; WBC, white blood cell count; RBC, red blood cell count; HGB, hemoglobin; HCT, hematocrit; MCV, mean corpuscular volume; MCH, mean corpuscular hemoglobin; MCHC, mean corpuscular hemoglobin concentration; RDW-CV, red cell volume distribution width-coefficient of variation; MPV, mean platelet volume; PCT, plateletcrit; PDW, platelet distribution width; dNLR, derived neutrophil to lymphocyte ratio; PLR, platelet-to-lymphocyte ratio; MLR, lymphocyte-to-monocyte ratio; NLR, neutrophil -to -lymphocyte ratio; SII, systemic immune-inflammation index; SIRI, systemic inflammation response index

**Note**: For categorical variables, frequencies and percentages are reported. For continuous variables, data are presented as either mean ± standard deviation (SD) for normally distributed data or median and interquartile range (IQR) for non-normally distributed data. P values < 0.05 were considered statistically significant and are denoted in bold

Although numerous hematological parameters have been identified as potential predictors of disease severity and prognosis in patients with COVID-19, there is a limited understanding of the hematological parameters associated with pulmonary infection by the Omicron variant of SARS-CoV-2. In addition, we initially compared the peripheral hematological parameters, and derived hematological profiles on admission after the onset of abnormal chest CT scans in the two groups to explore the underlying clinical predictive value. In total, 1342 COVID-19 patients with a normal chest CT scan on admission were enrolled as a control group. We analyzed the hematological parameters of the pulmonary infection group and the controls. Table 2 illustrates the markedly decreased counts of WBCs, NEUs, MONs, RBCs, PLT, HGB, HCT, PDW, dNLR, NLR, PLR, MLR, SII, and SIRI levels among COVID-19 patients with PI in contrast to the non-pulmonary infection group (p < 0.01). Notably, COVID-19 patients in the pulmonary infection (PI) group exhibited significantly higher levels of LYM counts, MCV, MCH, MCHC, RDW-CV, MPV, and PCT (p < 0.01). Additionally, when comparing the age distribution between the two groups, statistically obvious differences were observed in the < 18 years (2.21%), 18–60 years (30.34%), and ≥ 60 years (67.45%) age subgroups. Compared with non-PI individuals, PI individuals were older, and the majority had a lower level of systematic hematological parameters, which suggests that these hematological parameters have underlying clinical predictive value for developing a pulmonary infection in SARS-CoV-2 of Omicron cases. (Table 2). In short, the above results showed that the abnormality of hematologic parameters was associated with the presence of pneumonia in COVID-19 patients infected with the SARS-CoV-2 Omicron variant.

### Predictive performance of hematological parameters in the fever clinic for the diagnosis of COVID-19 patients infected with the omicron variant

**Fig. 3:**
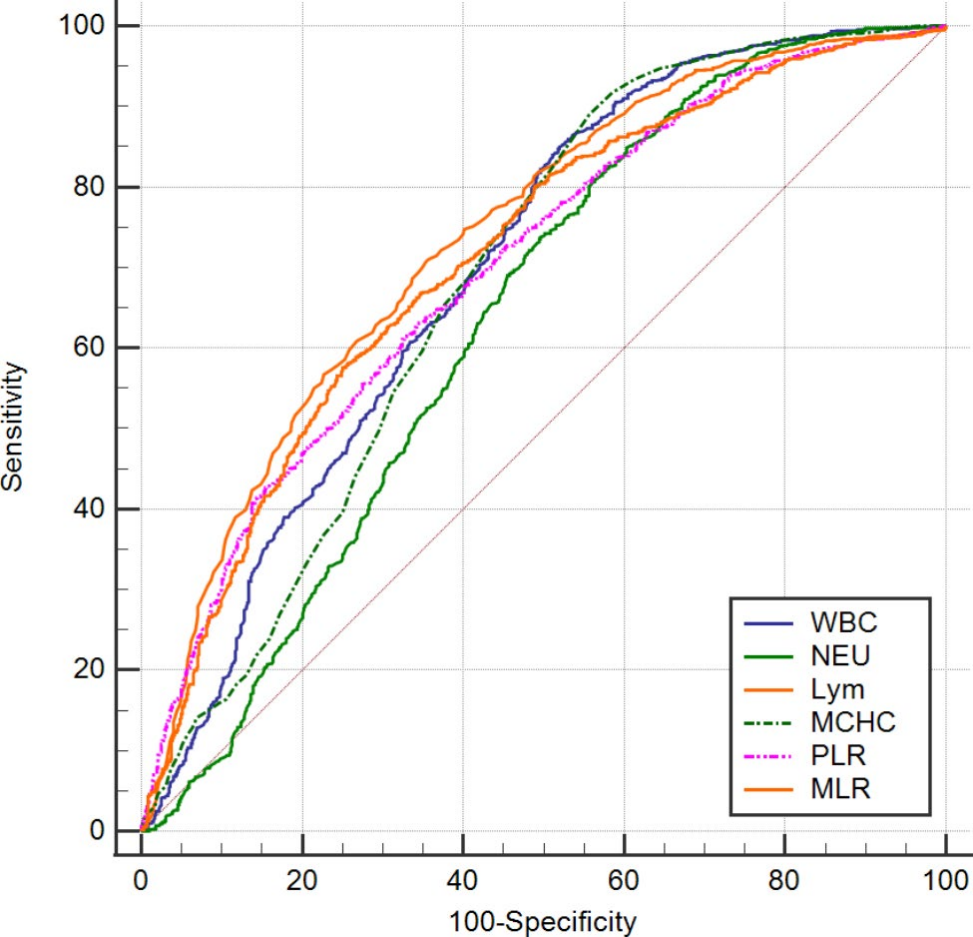
The receiver operating characteristic (ROC) curve analysis of hematological parameters to differentiate COVID-19 patients with fever from SARS-CoV-2 negative patients with fever. **Abbreviations**: WBC, white blood cell; NEU, neutrophils; Lym, lymphocytes; MCHC, mean corpuscular hemoglobin concentration; PLR, platelet-to-lymphocyte ratio; MLR, monocyte-to-lymphocyte ratio

Table 3; Fig. 3 illustrate the receiver-operating characteristic (ROC) curves we employed to evaluate the predictive value of hematological parameters for detecting Omicron variant SARS-CoV-2 infection among fever clinic patients. Compared with patients who tested negative for SARS-CoV-2 but presented with fever, the ROC curves for area under the curve (AUC) showed that LYM counts, MLR, PLR, WBC counts, MCHC, and NEU counts on admission were the top 6 hematological indicators with AUC values of 0.738 (95% Cl, 0.718–0.756, p < 0.01), 0.713 (95% CI, 0.693–0.732, p < 0.01), 0.702 (95% CI, 0.681–0.721, p < 0.01), 0.700 (95% Cl, 0.680–0.720, p < 0.01), 0.687 (95% Cl, 0.666–0.707, p < 0.01), and 0.637 (95% CI, 0.616–0.658, p < 0.01), respectively. Other hematological markers’ predictive performance between COVID-19 patients with fever and SARS-CoV-2 negative patients with fever is detailed in Table 3.

**Table 3:** Predictive performance of hematological parameters in the fever clinic for the diagnosis of COVID-19 patients infected with the Omicron variant ^a^

Variables	AUC	Cut-off value	Sensitivity (%)	Specificity (%)	95% CI	P -value
WBC, ×10^9^/L	0.700	≤ 8.37	84.95	48.08	0.680–0.720	< 0.01
NEU, ×10^9^/L	0.637	≤ 6.48	80.92	43.68	0.616–0.658	< 0.01
LYM, 10^9^/L	0.738	≤ 0.97	70.94	64.42	0.718–0.756	< 0.01
MON, ×10^9^/L	0.552	≤ 0.59	63.56	46.70	0.530–0.573	0.01
RBC, ×10^12^/L	0.539	> 4.82	36.51	71.43	0.517–0.561	0.01
HGB, g/L	0.533	> 138	45.08	63.46	0.511–0.554	0.01
HCT, %	0.596	> 41.3	45.50	70.05	0.574–0.617	< 0.01
MCV, fl.	0.594	> 87	65.20	50.27	0.572–0.615	< 0.01
MCHC, g/L	0.687	≤ 344	88.97	44.37	0.666–0.707	< 0.01
RDW-CV, %	0.533	≤ 12.8	71.31	35.99	0.511–0.555	0.01
Platelets, ×10^9^/L	0.592	≤ 249	85.77	27.75	0.571–0.614	< 0.01
MPV, fl.	0.555	> 15.8	63.79	45.88	0.533–0.576	< 0.01
PCT,%	0.563	≤ 20.5	74.66	34.75	0.541–0.584	< 0.01
PDW, %	0.555	> 15.8	63.79	45.88	0.533–0.576	< 0.01
dNLR	0.552	> 1.77	83.68	27.34	0.530–0.573	< 0.01
NLR	0.598	> 3.89	70.94	44.78	0.577–0.619	< 0.01
PLR	0.702	> 223.26	61.40	67.31	0.681–0.721	< 0.01
MLR	0.713	> 0.65	57.53	75.00	0.693–0.732	< 0.01
SII	0.563	> 871.84	63.56	47.12	0.542–0.585	< 0.01
SIRI	0.568	> 1.62	77.72	33.93	0.546–0.589	< 0.01

^a^ Abbreviations: AUC, area under curve; CI, confidence intervals

### Predictive performance of hematological parameters for the diagnosis of omicron variant infected COVID-19 patients with pulmonary infection

**Fig. 4:**
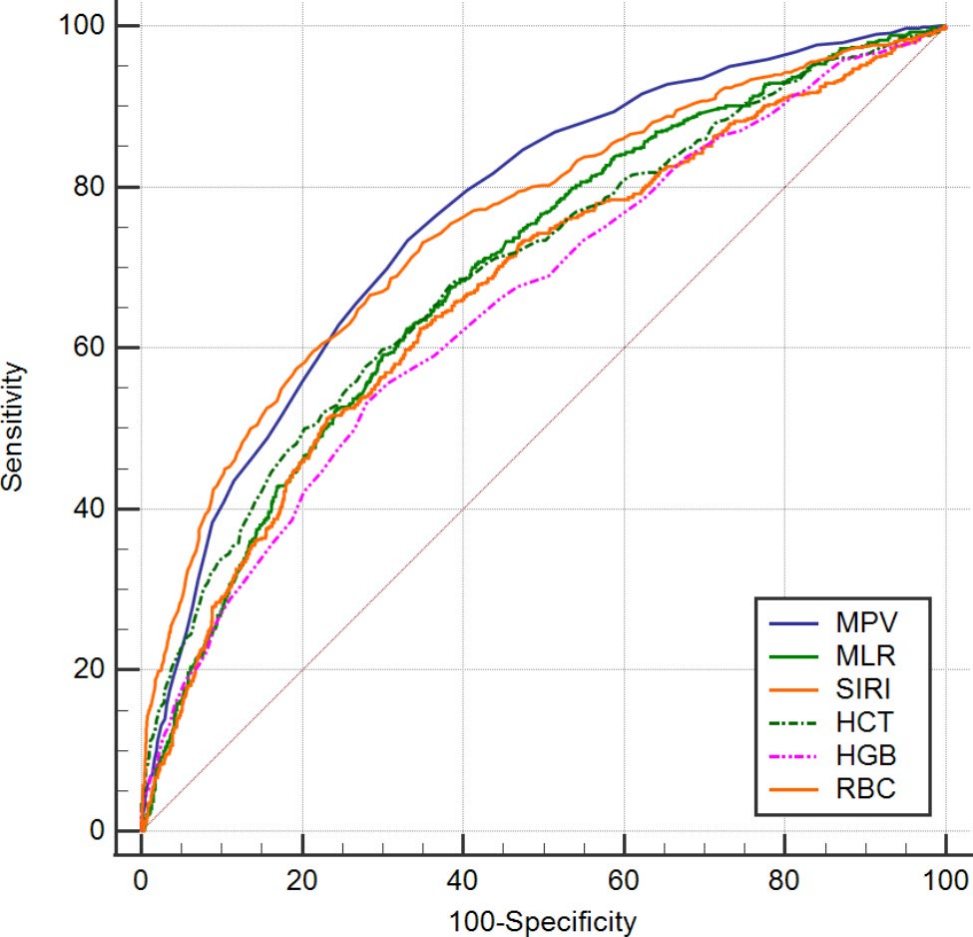
The receiver operating characteristic (ROC) curve analysis of hematological parameters to differentiate COVID-19 patients with pulmonary infection from without pulmonary infection. **Abbreviations**: MPV, mean platelet volume; MLR, monocyte-to-lymphocyte ratio; SIRI, systemic inflammation response index; HCT, hematocri; HGB, hemoglobin; RBC, red blood cell count

As shown in Table 4; Fig. 4, ROC curves were further performed to compare the predictive value of hematological parameters for predicting COVID-19 Omicron variant infected patients with PI. Compared with the COVID-19 patients without PI, the ROC curves of the AUC revealed that the MPV, RBC counts, HCT, MLR, SIRI, and MCH on admission, were the best top 6 hematological indicators, 0.764 (95% Cl, 0.745–0.782, P < 0.01), 0.753 (95% CI, 0.734–0.772, p < 0.01), 0.698 (95% CI, 0.677–0.718, p < 0.01), 0.694 (95% Cl, 0.674–0.714, p < 0.01), 0.673 (95% Cl, 0.652–0.694, p < 0.01) and 0.676 (95% CI, 0.655–0.696, p < 0.01), respectively. The predictive performance of other hematological markers for distinguishing between COVID-19 patients with PI and those without PI was detailed in Table 4. Overall, our findings suggested that routine hematological data on admission may serve as promising predictors for the early diagnosis of patients infected with the Omicron variant of COVID-19, and those with pulmonary infection.

**Table 4:** Predictive performance of hematological parameters for the diagnosis of Omicron variant infected COVID-19 patients with pulmonary infection

Variables	AUC	Cut-off value	Sensitivity (%)	Specificity (%)	95% CI	P value
WBC, ×10^9^/L	0.576	≤ 5.1	47.42	67.06	0.554–0.598	< 0.01
Neu, ×10^9^/L	0.600	≤ 3.72	51.55	66.47	0.578–0.621	< 0.01
Lym, 10^9^/L	0.620	> 0.700	70.25	50.75	0.598–0.641	< 0.01
Mon, ×10^9^/L	0.608	≤ 0.38	40.21	76.08	0.587–0.630	< 0.01
RBC, ×10^12^/L	0.753	≤ 4.28	59.79	78.61	0.734–0.772	< 0.01
HGB, g/L	0.658	≤ 128	53.31	72.06	0.637–0.678	< 0.01
HCT, %	0.698	≤ 38.8	59.94	70.12	0.677–0.718	< 0.01
MCV, fl.	0.645	> 88.2	75.85	45.23	0.624–0.666	< 0.01
MCH, pg	0.676	> 30.4	61.86	63.41	0.655–0.696	< 0.01
MCHC, g/L	0.605	> 338	49.19	65.20	0.583–0.626	< 0.01
RDW-CV, %	0.610	> 12.6	57.58	61.85	0.588–0.631	< 0.01
Platelets, ×10^9^/L	0.533	≤ 148	32.25	81.80	0.511–0.555	< 0.01
MPV, fl.	0.764	> 9.7	73.37	66.92	0.745–0.782	< 0.01
PCT,%	0.564	> 22.9	33.28	87.63	0.542–0.585	< 0.01
PDW, %	0.552	> 15.1	73.96	0.52	0.530–0.574	0.01
dNLR	0.610	≤ 3.16	65.68	55.81	0.588–0.631	< 0.01
NLR	0.638	≤ 5.75	69.07	56.04	0.616–0.659	< 0.01
PLR	0.630	≤ 218.87	58.23	62.74	0.609–0.651	< 0.01
MLR	0.694	≤ 0.55	62.30	67.06	0.674–0.714	< 0.01
SII	0.635	≤ 1080.18	67.60	54.92	0.614–0.656	< 0.01
SIRI	0.673	≤ 1.66	51.25	76.97	0.652–0.694	< 0.01

^a^Abbreviations: AUC, area under curve; CI, confidence intervals;

### Correlation analysis between fever days and hematological parameters in Omicron variant infected COVID-19 patients with pulmonary infection

The correlation between the number of fever days and hematological parameters was summarized in Table 5. We observed a positive correlation between the number of fever days and a hematological parameter MCHC (p = 0.01); Meanwhile, a statistically significant negative relationship was observed between the number of fever days and WBC counts (p = 0.03), RDW-CV (p = 0.01), Neutrophils counts (p = 0.01), dNLR (p < 0.01), NLR (p < 0.01), PLR (p = 0.01), SII (p = 0.01), and SIRI (p = 0.01). Other hematological parameters did not reach statistical significance (p > 0.05).

**Table 5:** Correlation of between fever days and hematological parameters in Omicron variant infected COVID-19 patients with pulmonary infection

Variables	Fever days	
	Correlation coefficient (r)	*P -value*
WBC counts, ×10^9^/L	-0.082	**0.03**
NEU counts, ×10^9^/L	-0.099	0.01
LYM counts, 10^9^/L	0.046	0.23
MON counts, ×10^9^/L	0.011	0.78
RBC counts, ×10^12^/L	0.034	0.38
HGB, g/L	0.066	0.09
HCT, %	0.047	0.22
MCV, fl.	0.032	0.41
MCH, pg	0.067	0.08
MCHC, g/L	0.097	**0.01**
RDW-CV, %	-0.100	**0.01**
Platelet counts, ×10^9^/L	0.033	0.38
MPV, fl.	0.024	0.53
PCT,%	0.031	0.41
PDW, %	-0.007	0.85
dNLR	-0.127	**0.01**
NLR	-0.132	**0.01**
PLR	-0.095	**0.01**
MLR	-0.054	0.16
SII	-0.108	**0.01**
SIRI	-0.105	**0.01**

**Note**: P values < 0.05 were considered statistically significant and are denoted in bold

## Discussion

Our study included 2,070 patients in the fever clinic, of which 1,342 had COVID-19 due to the SARS-CoV-2 Omicron variant. These patients exhibited lower levels of hematological parameters, including WBC counts, Neutrophil counts, Lymphocyte counts, Monocyte counts, MCHC, RDW-CV, and Platelet counts. In contrast, they had higher levels of RBC counts, HGB, HCT, MCV, and MCH compared to patients without COVID-19 infection. Derived hematological profiles such as dNLR, NLR, PLR, MLR, SII, and SIRI were significantly higher in the COVID-19 infection group than in the non-COVID-19 infection group. Upon performing ROC analysis, we demonstrated that LYM counts, NEU counts, MLR, PLR, WBC counts, and MCHC demonstrated superior diagnostic value and can serve as initial prescreening indicators for early COVID-19 diagnosis in fever clinics. Although the pathogenicity of the Omicron variant is greatly attenuated compared to previous strains, this does not imply that the Omicron variant has no clinical importance. Research on the value of hematological parameters in pulmonary infection (PI) patients at high risk of Omicron infection remains largely unexplored. Therefore, to fill this research gap, our study included 2,021 COVID-19 patients infected with the SARS-CoV-2 Omicron variant, of whom 679 had the presence of pulmonary infection. Patients with COVID-19 who had pneumonia exhibited lower levels of hematological parameters, including WBC counts, Neutrophils counts, Monocyte counts, RBC counts, HGB, HCT, and Platelet counts. Moreover, derived hematological profiles such as dNLR, NLR, PLR, MLR, SII, and SIRI were significantly lower in the PI group than in the non-PI group. Conversely, they had higher levels of Lymphocytes count, MCV, MCH, MCHC, RDW-CV, and MPV compared to patients without PI. Ultimately, the ROC analysis confirmed that MPV, RBC counts, HCT, MLR, MCH, and SIRI were more helpful than other hematological parameters in early identification of pneumonia in COVID-19 patients infected by the Omicron variant. To sum up, our study findings demonstrated that we performing rapid, inexpensive, and accessible routine hematological markers can improve the prescreening of COVID-19 patients infected by the Omicron variant. More importantly, these markers can assist in detecting individuals at risk of pneumonia, thereby providing clinicians with the opportunity for early intervention to prevent the onset of pneumonia.

Despite significant advances in the development of antiviral drugs and the widespread vaccinations, Omicron variants with highly mutated regions, resulting in enhanced transmissibility and immune evasion, have spread rapidly in many countries and pose a threat to global public health. Numerous studies have shown that the Omicron variant of SARS-CoV-2 primarily affects the upper airways, leading to flu-like symptoms with reduced pathogenicity [32–36]. Previous research has indicated that hematological parameters can serve as useful prognostic markers in COVID-19 patients [37]. Nevertheless, on the one hand, small sample-scale previous studies have compared the hematological parameters of Omicron variant-infected patients with fever and SARS-CoV-2 negative patients with fever [38]. More importantly, few studies have investigated the potential role of hematological parameters in the early identification of the Omicron variant in COVID-19 patients with pulmonary infection. Given the strengthened transmission and ability of the Omicron variant to evade the immune system, timely and effective diagnostics remain essential.

In this study, 2021 COVID-19 patients were enrolled, the majority of whom presented with an upper respiratory tract infection with symptoms (66.40%). Furthermore, febrile COVID-19 patients are characterized by a high dominant of females (54.25%) aged 18–60 years (55.51%), similar to previous studies [38, 39]. Additionally, the present study’s overall case-lung infection rate was approximately 33.60%. Most lung infection cases were observed in elderly patients, with prominent males at 57.29%, which may be because such patients have an increased risk of chronic lung diseases or a history of smoking, increasing expression levels of the angiotensin-converting enzyme 2 (ACE-2) receptor in these patients’ airways to develop pneumonia easily [40].

Firstly, because routine blood examination is required for the initial clinical examination in both the fever clinic and hospitalization, which strongly reflects infection disease severity and immune status in vivo, they are always used as prescreening predictors for developing their underlying clinical predictive value for many acute and chronic diseases. Secondly, our research group has previously demonstrated that routine hematological parameters can serve as reliable predictors to assess the severity of COVID-19 in patients infected with the wild-type variant [41]. For those reasons, We preferentially analyzed peripheral hematological parameters and their derived hematological profiles on admission in COVID-19 patients, with or without pulmonary infection, infected with the SARS-CoV-2 Omicron variant. We aim to assess whether previously valued hematological indicators retain their significance in evaluating the severity of COVID-19 patients in the era dominated by the SARS-CoV-2 Omicron variant.

Since the 2019 outbreak of the novel SARS-CoV-2 and through its subsequent variants, up to the current post-pandemic era, RT-qPCR tests have remained the gold standard for detecting COVID-19 and monitoring clinical samples for the presence of the virus [42]. However, the RT-qPCR test is associated with some limitations, such as lengthy turnaround times, high false-negative rates, and elevated costs [43]. Therefore, serological testing has emerged as a complementary method to RT-qPCR for more rapid diagnosis. With changes in China epidemic prevention policies since December 2022, convenient serological tests, primarily referring to COVID-19 antigen testing, have become widely used for self-testing at home. However, in view of insufficient sample collection, contamination of antigen test kits, and non-standardized procedures can lead to false-negative antigen test results. More importantly, antigen test results do not reflect the viral load or the severity of the disease [44]. In the context of current COVID-19 pandemic era, we require timely, inexpensive, and universally applicable biomarkers that reflect disease severity on admission. Thus, it is crucial to assess the value of hematological indicators for early differential diagnosis in high-risk individuals with pneumonia in COVID-19 patients infected with the SARS-CoV-2 Omicron variant, and they facilitate rational allocation of healthcare resources and accurate treatment strategies.

Previous studies have reported that COVID-19 febrile patients have lower leukocyte indices, including WBC counts, NEU counts, LYM counts, MON counts, and platelet counts, as well as higher levels of erythropoietic parameters, such as RBC counts, HGB, and HCT, compared to SARS-CoV-2 negative patients with fever, which is in line with our study [45–47]. This could be due to the fact that COVID-19 patients with fever tend to have higher viral loads, which can potentially impair their immune systems and hematological functions. Hematological abnormalities are most common in COVID-19 patients, particularly lymphopenia. It is generally believed to be an adaptive immune response of the host to viral infection or cytokine storms, affecting clinical outcomes and prognosis [48]. COVID-19 patients showed lymphopenia that may be caused by the release of monocytic reactive oxygen species (ROSs) via DNA damage, which leads to T-cell apoptosis. Furthermore, chronic infection with SARS-CoV-2 causes natural killer cells and T cells to become exhausted, resulting in a decreased lymphocyte count, which offers potential mechanisms for SARS-CoV-2-induced lymphopenia [49, 50]. Of note, our study unveiled a notable finding that COVID-19 patients with pulmonary infections showed a marked elevation in lymphocyte counts compared to those without lung infections, which is an interesting finding. We speculate that this may be attributed to the fact that lung infections often occur in the later stages of virus infection, when the viral load has already decreased and the immune response is more effective. As a consequence, the immune system tends to maintain normal levels of immune cells or even increase their numbers in response to the infection, which may explain the observed increase in lymphocyte count among COVID-19 patients with pulmonary infections. Neutrophilia has been observed in severe cases of COVID-19 for poor prognosis predictors [51]. Furthermore, previous studies have confirmed the characteristics of leukocyte indexes in Omicron patients with a lung infection. The expression levels of WBC counts and NEU counts decreased compared with those in the negative group [52], in line with our studies, and were associated with the attenuated pathophysiology of Omicron variants. Investigators found that similar to other coronaviruses, SARS-CoV-2 enters the host cell through angiotensin-converting enzyme 2 (ACE2) to facilitate viral fusion [53]. Thrombocytopenia is common occurrence among severe and critical COVID-19 patients, and mechanistic studies further suggested that direct activation of platelets by the SARS-CoV-2 virus, along with the potentiation of their prothrombotic function and inflammatory response via Spike/ACE2 interactions [54]. Similarly, previous studies have identified that low platelet counts may be a marker of disease severity in the Delta variant of SARS-CoV-2 and may contribute to determining the severity in Delta-infected patients [55]. NLR, PLR, and MLR are blood-based biomarkers that have been extensively studied as prognostic indicators for various immune system and diseases, including community-acquired pneumonia, sepsis, cardiovascular disease, and solid malignancies. Moreover, these hematological derived indices indicate systemic inflammation and may be associated with COVID-19 severity [58–62]. In addition, previous studies have proven that that COVID-19 severity and in-hospital mortality were significantly associated with systemic inflammation index, including the NLR, dNLR, PLR, MLR, SII, and SIRI [24, 41, 56–58]. Our study also discovered that, among COVID-19 patients infected with the SARS-CoV-2 Omicron variant, levels of dNLR, NLR, PLR, MLR, SII, and SIRI were all significantly all increased in cases with fever; conversely, these levels were significantly decreased in the presence of pneumonia. This result also suggests that these systemic inflammation indices may be considered as potential indicators for the diagnosis and progression assessment of COVID-19 Omicron variant infected patients. Novel systemic inflammation indexes called the systemic inflammatory response index (SIRI) and systemic immune-inflammation index (SII) based on peripheral lymphocyte, neutrophil, and platelet counts have been considered inexpensive and easily accessible markers confirmed to play an essential role in the diagnosis of cancer and infectious diseases [59]. Systemic inflammation markers, such as SIRI and SII, have been identified in COVID-19 patients and are useful for assessing inflammatory response and predicting in-hospital mortality [24]. Our study showed a significant increase in SII and SIRI levels among COVID-19 patients in comparison to individuals with fever but tested negative for SARS-CoV-2. Conversely, a significant decline was observed among COVID-19 patients with pulmonary infection. It may be due to the toxicity of Omicron was attenuated compared with that of the original strain and other mutants. Additionally, the weakened immune status of pneumonia patients and the suppressed inflammatory response, consistent with earlier publications [38, 52].

Furthermore, the ROC curve analysis in the present study observed that a decreased levels of LYM counts (0.738) on admission might be the most valuable hematological parameter for early diagnosis of COVID-19 with fever, followed by the decreased NEU counts (0.718), increased MLR (0.713), increased PLR (0.702), decreased WBC counts (0.700), and decreased MCHC (0.687), which is in concordance with previous report [38]. These are accessible and affordable hematological parameters that may assist clinicians in making timely differential diagnoses between COVID-19 patients and non-COVID-19 patients with fever. Additionally, another ROC curve showed that increased MPV (0.764) might be the most valuable hematological parameter, followed by decreased RBC counts (0.753), decreased HCT (0.698), decreased MLR (0.694), decreased SIRI (0.673), and increased MCH (0.676), for the early diagnosis of the presence of pulmonary infection in COVID-19 patients infected with the SARS-CoV-2 Omicron variant, which is not in accordance with previous literature [60]. This may be explained by differences in criteria for admission and discharge between different institutions, and variations in timing within the pandemic. Various factors can influence the results. A study also demonstrated that the mean platelet volume (MPV) might be used as an auxiliary test in predicting mortality in COVID-19 patients [61]. As far as we know, this is the first large-scale retrospective study that compares and assesses the predictive values of routine hematological parameters among patients infected with the COVID-19 Omicron variant with pulmonary infection versus those without pulmonary infection.

Additionally, a recent study showed that patients with pneumonic COVID-19 had elevated levels of white blood cells, neutrophils, and monocytes, and increased derived hematological markers such as NLR and MLR, compared to patients presenting with non-pneumonic Omicron COVID-19 infection; these results are contrary to our findings [60]. We speculate that these differences could stem from variations in disease stages and viral loads among the studied cohorts. In our control group, which did not show pulmonary infection, patients primarily came from fever clinics. They displayed short durations of fever, were predominantly in the early stages of the disease, and had high viral loads. In contrast, our cohort with pulmonary infections had longer fever durations and maintained comparatively lower viral loads. Furthermore, previous studies have highlighted that the Ct value of SARS-CoV-2, as determined by RT-qPCR on admission, can not only serve as an indicator of the viral load in COVID-19 patients but also act as an independent predictor for disease progression. Febrile COVID-19 patients exhibit a higher viral load compared to their non-febrile counterparts [38, 62]. Given its clinical significance, viral load should ideally be incorporated into our research analysis. Regrettably, changes in China’s epidemic prevention policy since December 2022 resulted in an unprecedented increase in Omicron cases. To improve clinical diagnosis and treatment during the period, our hospital reported only nucleic acid qualitative results for COVID-19, leaving us without specific Ct values. Despite our attempts to carefully retrieve the original data from each patient’s medical records, the nucleic acid tests, conducted in a provisional gas modeling laboratory, led to the irreversible loss of the data.

Notably, our study is based on the analysis of hematological data of each patient on admission. However, it cannot guarantee that all enrolled COVID-19 patients experienced disease onset at the same time. Individual heterogeneity, such as differences in the number of fever days and viral loads in vivo, may affect the blood parameter values of each patient on admission, thereby affecting the reliability of the findings. Therefore, we analyzed the correlations between fever days and all hematological parameters on admission in COVID-19 patients infected with the Omicron variant who had pulmonary infections. We found that eight parameters reached statistical significance. However, although these correlations were statistically significant, the Pearson correlation coefficients (R) for all parameters were low and very close to 0, indicating very weak correlations. This suggests that the relationship between the number of days each patient had a fever and these hematological parameters in our study is negligible. Based on these results, we can preliminarily determine that the correlation between the number of febrile days and the hematological parameters is not actually clinically significant, indicating that the results of our study may not be affected by individual heterogeneity. A decrease in Omicron’s virulence and a high vaccination campaign are the main reasons for the attenuation of symptoms in Omicron. This reminds us that many factors inside and outside vivo can influence hematological parameters, so they should be combined with history, clinical symptoms, and CT techniques for clinical diagnosis.

## Limitations

The current study has some limitations, which should be noted. Firstly, our study was a single-center retrospective study, which may not provide a comprehensive representation of the global population affected by the Omicron variant. This may affect the generalizability of our findings due to limitations of geographical and demographic biases. Secondly, the study was conducted during the outbreak of the Omicron variant in Jingzhou, Hubei, China, where most infected patients were mild and had a low risk of mortality, and only complete blood count (CBC) testing was available for analysis. Thirdly, the non-COVID-19 infected patients in the control group of this study were outpatients from fever clinics. Most of these patients exhibited mild symptoms, with fever being the initial symptom. Additionally, because epidemic prevention policies changed since December 2022, and fever patients would no longer be sent to designated isolation hospitals, doctors mainly ruled out the possibility of COVID-19 infection and did not proceed with specific etiological treatments, aiming to enhance population mobility. Most patients self-administered antipyretic drugs at home and did not seek further diagnosis or treatment at our hospital. Collectively, there is a lack of information on the proportion of other infectious diseases. Therefore, it is imperative that future studies focus on multi-center prospective studies and employ advanced statistical methods to better control for confounding factors and improve the accuracy of hematological parameters in terms of COVID-19 diagnosis and prognosis.

## Conclusions

In conclusion, our study found that most COVID-19 patients infected with the SARS-CoV-2 Omicron variant without the presence of pulmonary infection. In addition, our findings demonstrated that LYM counts, NEU counts, MCHC, WBC counts, MLR, and PLR markers can serve as potential prescreening indicators for early COVID-19 diagnosis in fever clinics. Moreover, MPV, RBC counts, HCT, MLR, MCH, and SIRI can predict the presence of pulmonary infection in COVID-19 patients infected with the SARS-CoV-2 Omicron variant. Therefore, our findings provided foresightful insights into these rapid, inexpensive and widely available biomarkers, indicating that assessing hematological parameters on admission has the benefit of enabling clinicians to make practically applied in a clinical setting and helping clinicians in guiding decision quickly in the early stages of hospital treatment. However, the results of this study require further validation and should be corroborated by conducting large-scale, multi-center prospective studies.

## Data Availability

The datasets generated and/or analysed during the current study are not publicly available due to privacy or ethical restrictions. but are available from the corresponding author on reasonable request.

## Abbreviations

SARS: CoV-2 Severe acute respiratory syndrome coronavirus 2
COVID: 19 Coronavirus disease 2019
PI: Pulmonary infection
ROC: Receiver operating characteristic curves
LYM: Lymphocyte
NEU: Neutrophil
MLR: Monocyte-to-lymphocyte ratios
PLR: Platelets-to-lymphocyte ratios
WBC: White blood cell
MCHC: Mean corpuscular hemoglobin concentration
AUC: Area under the curve
MPV: Mean platelet volume
RBC: Red blood cell
HCT: Hematocrit
MCH: Mean corpuscular hemoglobin
SIRI: Systemic inflammation response indexes
VOC: Variants of concern
WHO: World Health Organization
dNLR: derived neutrophil to lymphocyte ratio
SII: Systemic immune-inflammation index
NLR: Neutrophil to lymphocyte ratio
RT-qPCR: Reverse transcription quantitative PCR
SD: Standard deviation
IQR: Interquartile range
CI: Confidence interval
ACE2: Angiotensin-converting enzyme 2
CBC: Complete blood count
